# Anisotropic perception of slant from texture gradient: Size contrast hypothesis

**DOI:** 10.3758/s13414-015-1024-0

**Published:** 2015-11-18

**Authors:** Atsuki Higashiyama, Tadashi Yamazaki

**Affiliations:** Ritsumeikan University, Kyoto, Japan; Department of Psychology, Ritsumeikan University, Tojiin-kita-machi, Kita-ku, Kyoto 603-8346 Japan

**Keywords:** Slant perception, Anisotropy, Texture gradient, Motion gradient, Size contrast

## Abstract

When we see an optical pattern that has a gradient of the size and/or density of its texture elements, we often perceive a surface that is slanted in depth. Our inquiry was to ask whether the effect of a texture gradient depends on the direction of the gradient (ground, ceiling, and sidewall patterns) or on the position of the observer’s head (upward, forward, or downward). In Experiments 1 and 2, a total of 63 observers judged the apparent slant of polka-dot, grid, or flagstone patterns; regardless of head position, the ground patterns were judged to be closer to the frontal plane than were the other patterns. This means that there is a visual anisotropy in head-centric slant perception. To explain this result, we assumed accumulated positional effects of size contrast—that is, a tendency to perceive the size of the upper part of visual space to be larger than the size of the lower part. This hypothesis was examined in two subsequent experiments by reducing the size contrast among the texture elements. When 23 observers viewed regularly arranged same-sized-dot patterns with gradients of the interdot interval and with linear perspective of the dotted lines, anisotropic effects were still detected. When 22 observers viewed dynamic random-dot patterns with gradients of velocity, the anisotropic effects mentioned above were removed in many cases, and the *ceiling* patterns were sometimes judged to be less slanted than the other patterns. These results partially support the size contrast hypothesis and were compared with the predictions from other hypotheses.

Gibson (1950a, 1950b, 1979) maintained that an optical pattern generates a visual surface, and if the optical pattern has a texture gradient, the visual surface is a plane slanted in depth. By the *slant* of a plane, we mean the deviation from the frontal plane, which is a zero slant. Changes in the gradient would indicate changes in the slant, since the direction of the gradient indicates the direction in which the surface is receding from the observer, and the steepness of the gradient indicates the extent to which the surface is slanted from the frontal plane.

A note about several possible types of slants is needed here. Gibson (1950a) and Gibson and Cornsweet (1952) distinguished optical slant from gravitational (or geographical) slant. The former is the slant of a surface to the line of regard, and the latter is the slant of a surface to the horizontal and vertical axes determined by gravity. We did *not* use either of these slants in this study, but use the head-centric slant of a surface, which is determined by the Frankfort horizontal plane/line (i.e., a line running from the porion to the orbitale points on the skull) and a line that is perpendicular to it. If the head is held stationary, the head-centric slant is constant, independent of the direction of gaze. If and only if the head is gravitationally upright, the head-centric slant approximates the geographical slant, because the Frankfort horizontal plane approximates the ground plane in direction.

In this study, we investigated whether the slant of the plane generated by texture gradient changes with the direction of the gradient. See, for example, the upper left panel of Fig. 1, in which texture density increases regularly and gradually from bottom to top. Does the slant of this pattern appear to be the same as that of the inverted texture pattern shown in the upper right? How about other patterns in which the texture density increases from left to right or from right to left (lower panels of Fig. 1)? If visual space is homogeneous, as Gibson (1950a) might assume, the apparent slant of these patterns would be the same, regardless of the direction of the gradient; but if visual space is anisotropic for some reason, the apparent slant may depend on the direction of the gradient.

**Fig. 1:**
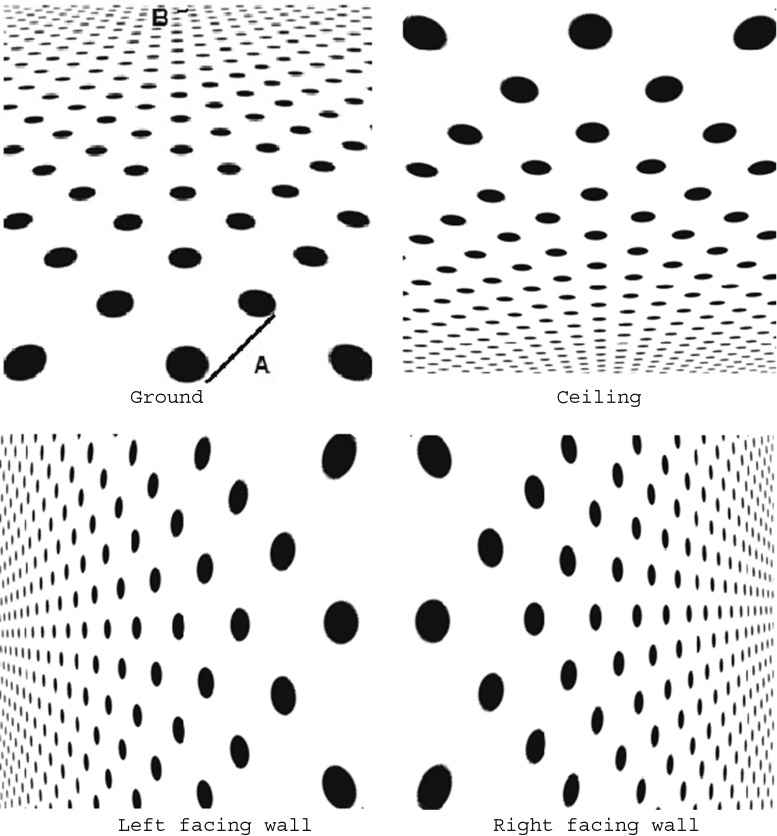
Polka-dot patterns. Upper left, ground; upper right, ceiling; lower left, left-facing wall; lower right, right-facing wall. These patterns have gradients of dot size, interdot interval, and dot density, and also have implicit linear perspective. The steepness of the gradients here is 1.43 for all patterns

The line of research that Gibson began has led to an increasingly detailed analysis of way that texture gradient underlies slant perception. A brief summary regarding *absolute* slant judgments is as follows. (1) Judged slant is greater for a texture of regular rectangles than for a texture of irregular grass-like prints (Gibson, 1950a). Also, regular patterns of circles are judged to be more slanted than irregular cross-sectional patterns of stones (Norman, Crabtree, Bartholomew, & Ferrell, 2009). (2) Regularity in the size and shape of texture elements is more important than regularity in the distribution of the elements (Flock & Moscattelli, 1964). However, there is some evidence that random variations in the size and shape of texture elements have no detectable effect on judged depth of curved surfaces (Todd & Akerstrom, 1987). (3) There is an optimum texture density range, below which the texture elements may appear to be independent figures and above which they become fused (Gruber & Clark, 1956). (4) Contour convergence is usually more effective in judging slant than is a texture gradient (see the review in Braunstein, 1976, chap. 5). This idea is supported by the demonstrations by Stevens (1981) and by Todd and Akerstrom (1987, their Figs. 19 and 20). Indeed, the addition of a texture gradient to a figure with visible contours has little effect on judged slant (Clark, Smith, & Rabe, 1956). (5) When slanted surfaces are viewed through an aperture or a reduction screen, they appear closer to the frontal plane than when they are normally viewed (Norman et al., 2009). (6) When pictures of spatial layouts are enlarged in visual angle, the apparent depth of curved surfaces (Todd, Thaler, & Dijkstra, 2005; Todd, Thaler, Dijkstra, Koenderink, & Kappers, 2007) and the apparent depth between targets (Lumsden, 1983) in the pictures are smaller than when they are reduced. (7) Polar projection of a slanted surface with a texture makes the surface appear more slanted than the parallel projection (Tibau, Willems, Van den Bergh, & Wagemans, 2001; Todd & Akerstrom, 1987). (8) There is no prominent aging effect in slant perception (Norman et al., 2009).

The summary regarding *relative* slant judgments is as follows. (9) The perspective of texture elements is the most important source of information, and their compression (or form ratio) is also to some degree effective (Braunstein & Payne, 1969; Cutting & Millard, 1984; Knill, 1998; Vickers, 1971). (10) The difference threshold for slant decreases as a textured surface is slanted from the frontal plane (Hills, Watt, Landy, & Banks, 2004; Knill, 1998; Knill & Saunders, 2003; Rosas, Wichmann, & Wagemans, 2004). (11) The difference threshold for slant decreases with increasing horizontal field of view (Knill, 1998). (12) The difference thresholds are small for polka-dot patterns and large for noise patterns (Rosas et al., 2004).

Apart from studies of texture gradients, it is said that visual space is anisotropic. Anisotropy is used to mean that visual space has different properties in different directions; visual attributes such as size, angle, velocity, form, and brightness have anisotropic natures (Rock, 1973, p. 96). Examples of anisotropic size perception are the horizontal–vertical illusion (i.e., a vertical line appears longer than a horizontal line of the same physical size—Higashiyama, 1996; Luckiesh, 1922, chap. 4; Ninio, 1998/2001, chap. 3; Robinson, 1972, p. 96; Tolansky, 1964, chap. 2), the tendency to perceive the upper half of visual space to be larger than the lower half (Luckiesh, 1922; Robinson, 1972), the tendency to perceive an interval in depth to be shorter than an equal interval in the frontoparallel plane (Foley, 1968; Levin & Haber, 1993; Li & Durgin, 2013; Loomis, Da Silva, Fujita, & Fukushima, 1992; Loomis & Philbeck, 1999; Norman, Todd, Perotti, & Tittle, 1996; Toye, 1986; Wagner, 1985; Watanabe, 2004), and the moon illusion (i.e., the horizontal moon appears larger than the zenith moon—Hershenson, 1989; Ross & Plug, 2002).

It is not certain that there is visual anisotropy in slant perception. Gibson (1950b) actually failed to find it for the apparent slant of textured planes when he compared one plane with an upward gradient and one with a downward gradient of texture density. Nevertheless, a number of recent studies have suggested that possibly there is visual anisotropy in slant perception, although no study has directly measured the apparent slant of textured planes. McCarley and He (2000) required observers to segregate and search multiple items that were distributed in depth. When the stimulus displays appeared to recede top-away in depth (i.e., a ground-like display), the time to respond to targets in the displays was faster than when the displays appeared to recede bottom-away in depth (i.e., a ceiling-like display). Bian, Braunstein, and Andersen (2005, 2006) and Bian and Andersen (2008) have shown that observers have a preference to judge distance in accordance with optical contact provided by the ground surface, rather than by the ceiling or sidewall surface. It was also demonstrated that ground surfaces are superior to ceiling surfaces in binocular rivalry (Ozkan & Braunstein, 2009) and in detection of changes on the surface (Bian & Andersen, 2010). These studies suggest that objects are well organized on the ground surface. Furthermore, Meng and Sedgwick (2001, 2002) pointed out that objects on the ground are related to each other through nested contact relations among adjacent surfaces and that observers make use of this information in distance perception, but they did not refer to objects on the ceiling or sidewall surfaces.

It is also possible to expect that the apparent slant of a surface with a texture gradient would depend on the observer’s head position. If anything, this should be called *proprioceptive anisotropy*, in contrast with the visual anisotropy just mentioned above. It has been documented that the apparent size (Higashiyama, 1992, 1996; Ross & Plug, 2002), apparent distance (Higashiyama & Adachi, 2006), and brightness (Higashiyama & Toga, 2011) of objects change with the position of the head or trunk. Since an interval in depth appears to diminish when it is observed from between the legs (Higashiyama & Adachi, 2006), one might expect that if the head were leaned from the normal upright position, a surface with a texture gradient might appear less slanted than when it was observed normally.

In Experiment 1 of this study, we compared texture densities that increased from bottom to top (i.e., ground-like slants that appear top-away relative to the frontal plane) with texture densities that changed in the reverse direction (i.e., ceiling-like slants that appears bottom-away relative to the frontal plane). These patterns consisted of oval polka dots generated by computer or of rectangular flagstones taken by camera. These patterns may convey a strong impression of a plane being slanted, because, as we summarized above, they are regular in size, shape, and the distribution of texture elements, and they also produce linear perspective by connecting the texture elements perceptually. The observers viewed these patterns with the head upward, forward, or downward. Since we were interested in the apparent head-centric slant of surfaces, the observers were allowed to move their eyes freely, and the spatial relation of the stimulus display to the head was fixed, regardless of changes in head position. In Experiment 2, we used flagstone patterns again and compared ground, ceiling, and sidewall patterns. As a control, we examined grid patterns that were geometrically similar to the flagstone patterns.

As will be clear in the results of Experiments 1 and 2, the judged slants varied with the direction of the gradient, and this anisotropy occurred independently of head position. We interpreted this to be the result of the accumulated positional effects of size contrast between the texture elements (i.e., the higher that elements are in a display, the larger their size appears) and attempted to confirm this interpretation in the subsequent experiments. For this purpose, we reduced size contrast by holding the element size constant (Exp. 3) or by replacing the gradients of element size, interelement interval, and element density by the gradient of velocity (Exp. 4). We predicted that, by reducing the size contrast, the anisotropic slant effects might be weakened or eliminated.

## Experiment 1

### Method

#### Observers

The observers were 18 university students (nine males and nine female) with a mean age of 20.2 years.

#### Apparatus and visual patterns

Textured patterns were presented in the head-mounted display (HMD; Virtual-Eye HEWDD-768) with the weight of 1,950 gram-weight. A perfectly identical pattern was always presented straight ahead of each eye, which generated zero binocular disparity. The visual field was 140° horizontally and 90° vertically.

**Fig. 2:**
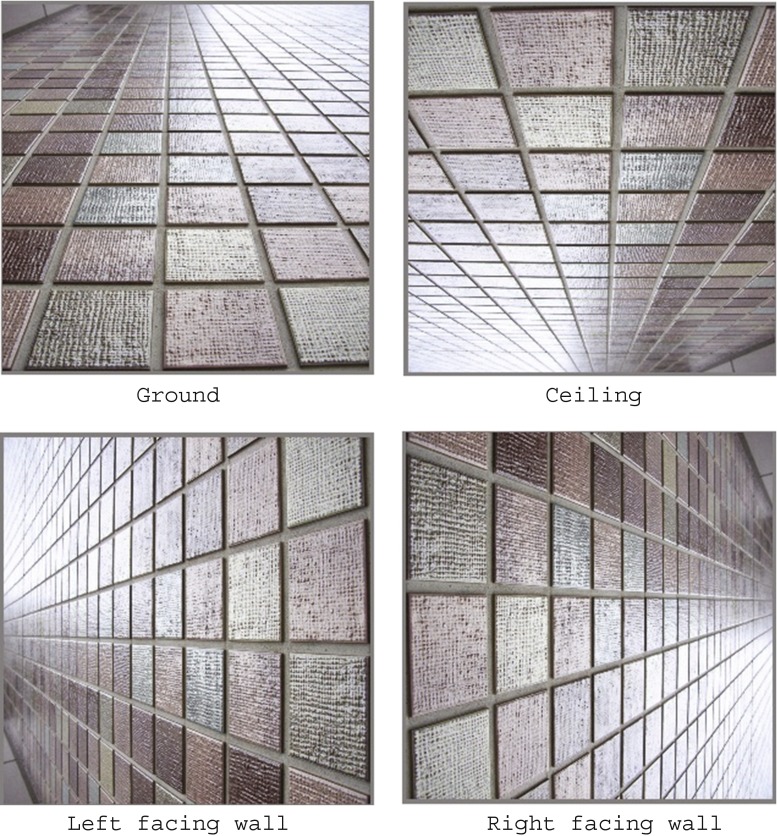
Flagstone patterns. Upper left, ground; upper right, ceiling; lower left, left-facing wall; lower right, right-facing wall. The steepness of the gradients here is 1.66 for all patterns

We used two types of textures: one, oval polka dots (Fig. 1), and other, rectangular flagstones (Fig. 2). Each textured pattern was 32.8° wide × 32.8° high in visual angle. Seven polka-dot patterns, which differed in the steepness of their gradients, were made with Transform in Photoshop from a regularly spaced circle pattern in which a black circle was placed at each intersection of the lines in an imagined square grid. Four flagstone patterns were also made by taking pictures of the sidewall of an actual building on the campus. The gradient was varied by changing the distance between the wall and the camera. Each pattern was presented so as to appear like the ground (i.e., lower density at the bottom of the picture and higher density at the top) or like the ceiling (i.e., higher density at the bottom and lower density at the top).

For convenience, the steepness of the gradient was defined as 2(*A* – *B*)/(*A* + *B*), where *A* is the largest center-to-center distance of diagonally adjacent texture elements and *B* is the smallest one (see the upper left of Fig. 1). The diagonal distance may be a composite of perspective gradient and compression gradient (Cutting & Millard, 1984) or of the perspective and form ratios (Braunstein, 1976). Our definition of the steepness of a gradient represents the range (*A* – *B*) of the composite relative to the mean (*A + B*)/2. The steepness values for the polka-dot patterns were 0.00, 0.12, 0.28, 0.48, 0.87, 1.23, and 1.43, whereas the steepness values for the flagstone patterns were 0.75, 1.05, 1.26, and 1.66.

**Fig. 3:**
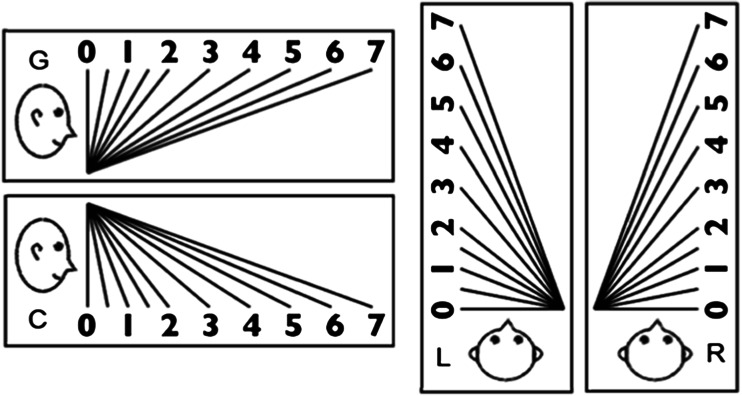
The G, C, L, and R scales, used to match the apparent slants of ground, ceiling, left-facing wall, and right-facing wall patterns

The G and C scales that are shown in Fig. 3 were vertically apposed 1.1° to the right of the textured pattern within the HMD. Each scale was 27.3° wide × 11.5° high in visual angle. The G scale was used for the ground pattern, and the C scale was used for the ceiling pattern. To make the observers understand the head-centric slant, we drew a schematic head next to each scale. In each scale, ten lines were drawn that were differently sloped relative to the head. The lines were nominally numbered from 0 to 7. The numbers “0,” “1,” “2,” “3,” “4,” “5,” “6,” and “7” corresponded to slants of 0°, 21.4°, 38.2°, 49.7°, 57.5°, 63.0°, 67.0°, and 70.0° from the frontal plane of the drawn person, respectively. Also, a “0.5” line was inserted between lines “0” and “1,” and a “1.5” line between lines “1” and “2.” Note that the angles between the adjacent lines in each scale decrease as the lines are slanted from the frontal plane. This is due to a small difference threshold for a plane slanted away from the frontal plane (Hills et al., 2004; Knill, 1998; Knill & Saunders, 2003).

#### Procedure

**Fig. 4:**
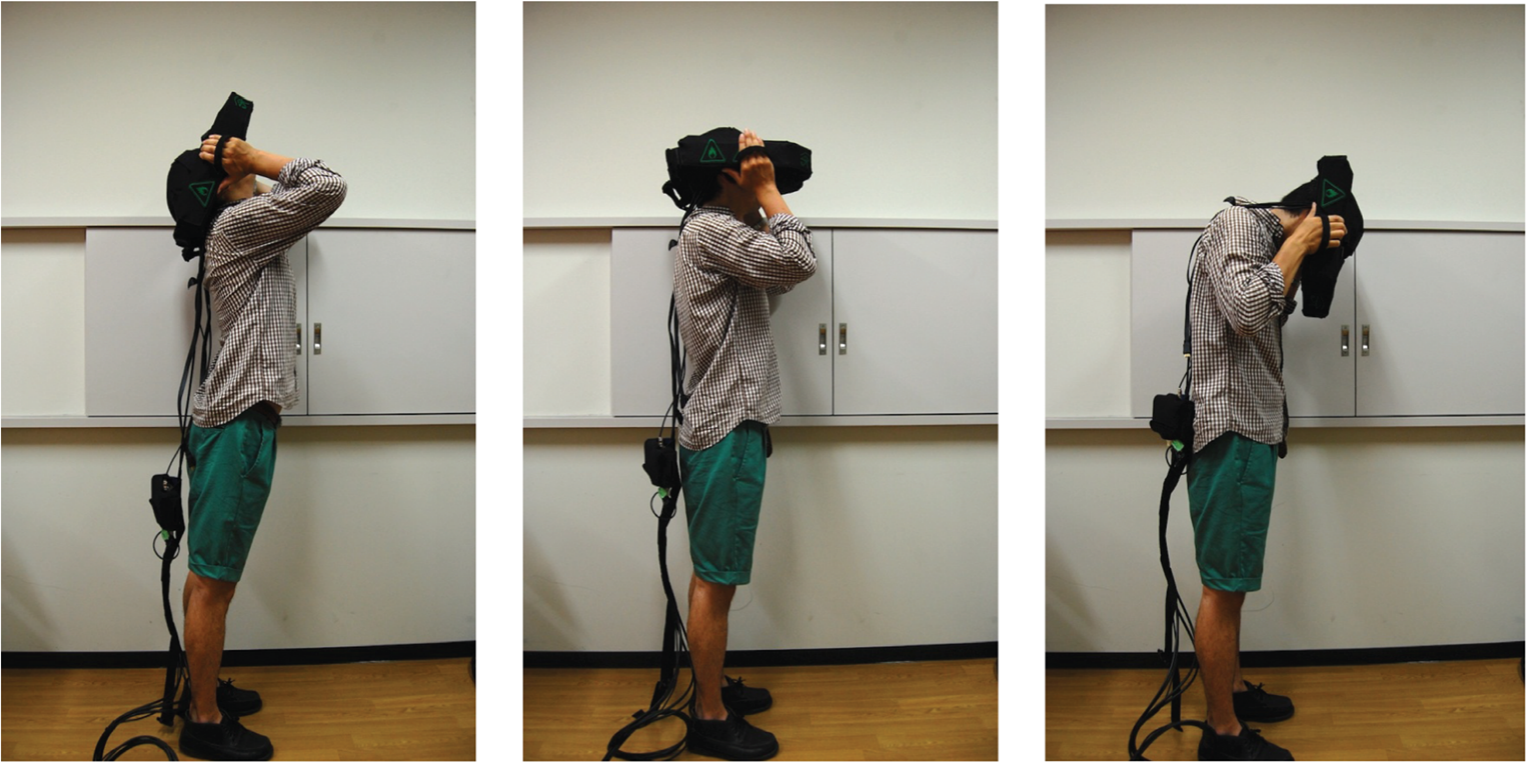
The head-mounted display: Left, upward viewing; center, forward viewing; right, downward viewing

Each observer wore the HMD and checked whether to smoothly rotate the head upward, forward, or downward (Fig. 4). After having made sure that the head was rotated normally, the observer viewed one pattern at a time binocularly under a given head position. In the upward head condition, the observer leaned the head back so as to look at the ceiling above the head; in the downward head condition, the observer leaned the head down so as to look at his/her instep; in the forward head condition, the observer directed the head to the horizontal line. In each head condition, the observer was asked which of the higher or lower density of each pattern was farther away and was then required to choose a line in the scale that was most suitably matched to the slant of the textured plane. When the slant of the textured plane was somewhere between adjacent scale lines, the observer was allowed to choose both lines. No time limit for judgments was imposed on the observers. After the observer had judged the slant of one pattern on each trial, the other pattern was exchanged for the next trial. Half of the observers judged the polka-dot patterns following the flagstone patterns, and the others judged the patterns in the reverse order. The order of head conditions was counterbalanced among observers. The combinations of steepness and direction were randomly presented to each observer under a given head condition.

### Results

#### Polka-dot patterns

**Fig. 5:**
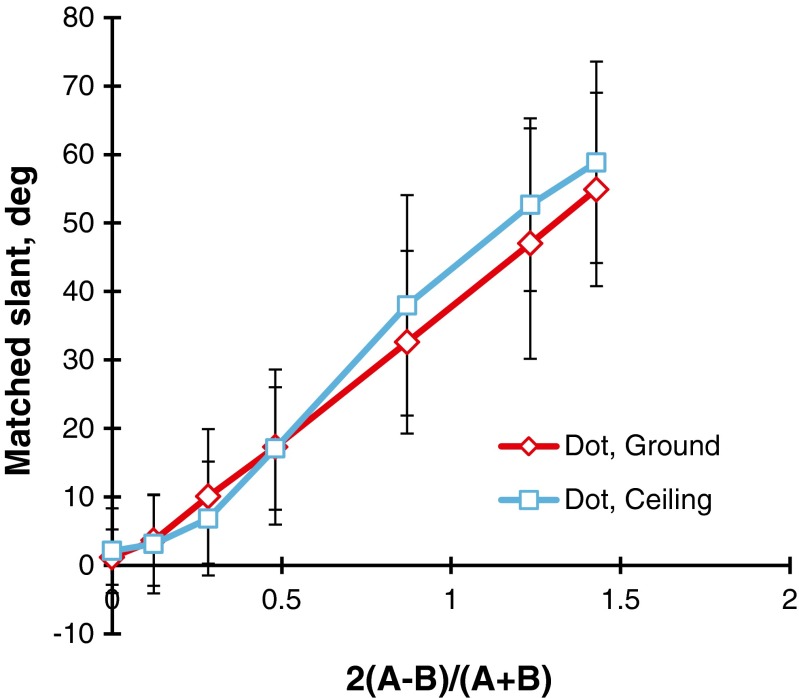
Mean matched slants in degrees for the polka-dot patterns, as a function of the steepness of the gradient. The parameter is the direction of the gradient (ground vs. ceiling). The bar attached to each point stands for one standard deviation on either side

The lines chosen by each observer were converted to slants in degrees. When two lines were chosen, the mean slant was taken for the statistical score. For the polka-dot patterns, a three-way (Steepness × Direction × Head) repeated measures analysis of variance (ANOVA) was done on the judged slants, which revealed that the main effect of steepness, *F*(6, 102) = 283.2, *p* < .001, and the Steepness × Direction interaction, *F*(6, 102) = 3.7, *p* < .01, were significant. The tests of the simple main effects indicated that for the steepness values of 0.87, 1.23, and 1.43, the mean judged slant of the ground pattern was significantly different from that of the ceiling pattern: *F*(1, 119) = 8.1, *p* < .01; *F*(1, 119) = 8.9, *p* < .001; and *F*(1, 119) = 4.3, *p* < .05, respectively. The paired mean judged slants of the ground and ceiling patterns were 32.6° versus 38.0°, 47.0° versus 52.7°, and 54.9° versus 58.9° for the steepness values of 0.87, 1.23, and 1.43, respectively. Figure 5 shows the mean judged slants as a function of the steepness of the gradient, with the direction of the gradient as the parameter. The main effect of head was not significant, and the interaction of head and the other source was also not significant.

#### Flagstone patterns

**Fig. 6:**
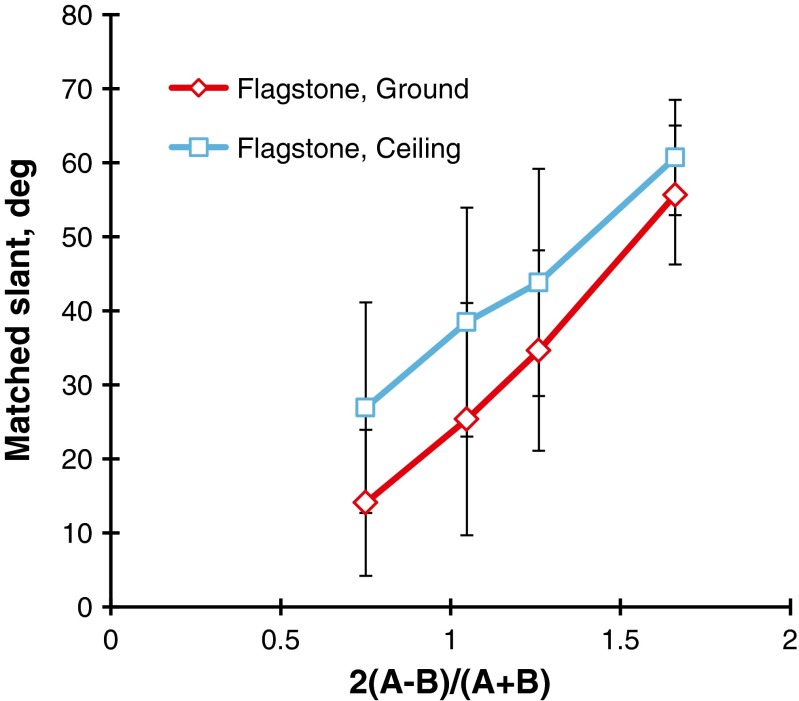
Mean matched slants in degrees for the flagstone patterns as a function of the steepness of the gradient. The parameter is the direction of the gradient (ground vs. ceiling). The bar attached to each point stands for one standard deviation on either side

Figure 6 shows the results of the flagstone patterns in the same way as the polka-dot patterns. A three-way repeated measures ANOVA revealed that the main effect of steepness, *F*(3, 51) = 125.3, *p* < .001, and the main effect of direction, *F*(1, 17) = 69.5, *p* < .001, were significant. This means that the mean judged slants of the ground patterns, which were 14.1°, 25.3°, 34.6°, and 55.6° with increasing steepness of the gradient, were consistently smaller than the corresponding mean judged slants of the ceiling patterns, 26.9°, 38.5°, 43.8°, and 60.7°. The Steepness × Direction interaction, *F*(3, 51) = 3.6, *p* < .05, was also significant. This means that the differences in mean judged slant between the ground and ceiling patterns were larger for a smaller steepness. The main effect of head was not significant, and the interactions of head and the other factors were not significant.

### Discussion

There are several new findings in this experiment. First, as is evident from Figs. 5 and 6, the mean judged slant was a linear increasing function of the steepness of the gradient. Second, the ground patterns were judged to be less slanted from the head-centric frontal plane than the ceiling patterns—in particular, for the steepness around unity. The anisotropic effect, which is represented by the difference in mean judged slants between the ground and ceiling patterns, was larger for the flagstone patterns than for the polka-dot patterns.

**Fig. 7:**
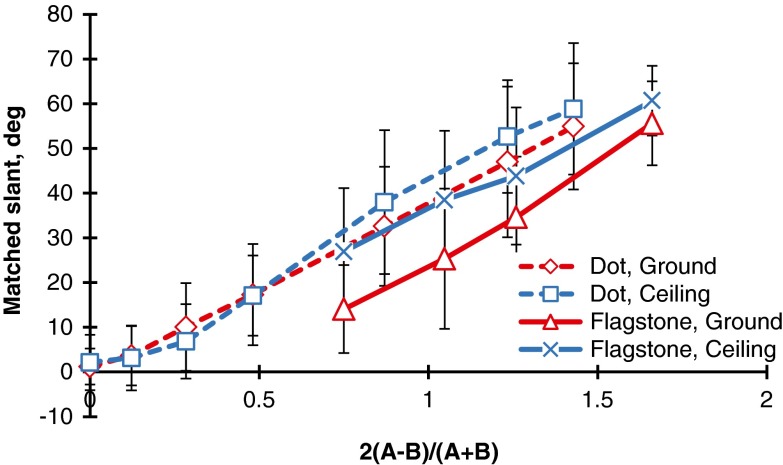
Comparison of the polka-dot and flagstone patterns. The bar attached to each point stands for one standard deviation on either side

Figure 7 compares the results for the polka-dot and flagstone patterns. It is interesting to note that the mean judged slants of the polka-dot patterns were larger than those of the flagstone patterns. A similar outcome was obtained by Gibson (1950a) and Norman et al. (2009), who indicated that the apparent slant of geometric element patterns like circles or rectangles is larger than that of natural textured surfaces like grass or stone. This problem was examined again in Experiment 2.

## Experiment 2a

The ground patterns appeared less slanted than the ceiling patterns. This was true regardless of the change in head position. This means that there is a visual, not a proprioceptive, anisotropy in slant perception. In Experiment 2a, the observers held their heads forward and judged not only the slants of ground and ceiling patterns, but also those of sidewall patterns.

### Method

#### Observers

The observers were 21 university students (eight males and 13 females) with a mean age of 19.2 years.

#### Apparatus and visual patterns

The apparatus was the same HMD used in Experiment 1, and the patterns were the same flagstone pictures used in Experiment 1. Each pattern was presented in four directions of gradient so that the density of the flagstones increased from bottom to top (ground), from top to bottom (ceiling), from right to left (left-facing wall), and from left to right (right-facing wall). The G and C scales were vertically apposed in the HMD 1.1° to the right of the ground or ceiling patterns; the L and R scales were laterally apposed 1.1° to the right of the sidewall patterns.

#### Procedure

After having worn the HMD, each observer held his or her head forward and viewed one pattern at a time binocularly. The observer was asked which of the higher or lower density in the pattern was farther away from the observer and was then required to choose either one line or two neighboring lines that were most suitably matched to the slant of the textured plane. The G, C, L, and R scales were used for the ground, ceiling, left-facing, and right-facing wall patterns, respectively. Each observer judged 32 (4 × 4 × 2) combinations of steepness, direction, and replication, which were randomly presented for each observer.

### Results

**Fig. 8:**
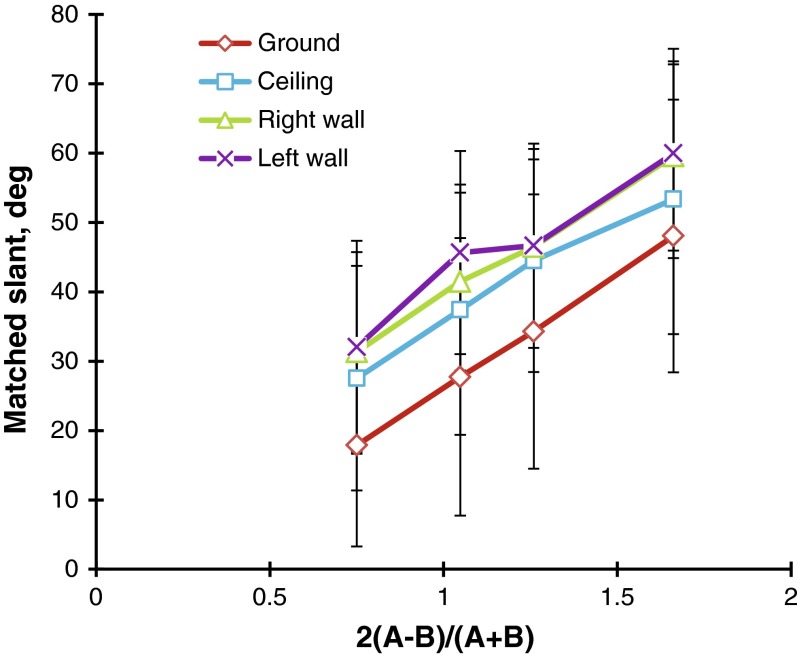
Mean matched slants in degrees for the flagstone patterns as a function of the steepness of the gradient. The parameter is the direction of the gradient: ground, ceiling, and left- and right-facing walls. The bar attached to each point stands for one standard deviation on either side

The observers’ judgments were converted into slants in degrees in the same way as in Experiment 1, and the two replications were averaged for the statistical scores. Figure 8 shows the mean judged slants taken over the 21 observers. A two-way (Steepness × Direction) repeated measures ANOVA revealed that the main effect of steepness, *F*(3, 60) = 59.0, *p* < .001, and the main effect of direction, *F*(3, 60) = 10.2, *p* < .001, were significant. Ryan’s multiple pairwise comparison tests indicated that the mean judged slant of the ground patterns was significantly different from those of the ceiling, *t*(60) = 3.1, *p* < .05, the left-facing wall, *t*(60) = 5.0, *p* < .05, and the right-facing wall, *t*(60) = 4.5, *p* < .05, patterns, respectively. *MSE* = 332.7 for all subordinate tests. There was no significant difference in the mean judged slants between the ceiling and sidewall patterns or between the different sidewall patterns.

### Discussion

These results suggest that the ground patterns were judged to be less slanted not only than the ceiling patterns, but also than the sidewall patterns. The differences in mean judged slants between the ground patterns and other patterns ranged from about 10° to 14°, mainly depending on the steepness of the gradient. This compares to the range of differences obtained for the flagstone patterns in Experiment 1 (about 5° to 13°).

## Experiment 2b

The anisotropy for the flagstone patterns was remarkable. This great effect may be due to the lightening as well as to the texture gradient, as is shown in Fig. 2. To control lightening, in this experiment, the flagstone patterns were replaced with grid patterns lacking a difference in lightening. It was also possible to assume that the anisotropic effect could be obtained only under a specific viewing condition (i.e., simultaneous viewing of the standard and matching stimuli in the HMD) or with a specific method of measurement (i.e., multiple choice). As a converging operation, in this experiment, the standard and the variable were compared successively, without the HMD, under natural viewing, and the variable was changed continuously by the method of adjustment.

### Method

#### Observers

The observers were 24 university students (12 males and 12 females) with a mean age of 23.5 years.

#### Apparatus and visual patterns

Either one standard pattern or a variable line was presented at a time in a Nanao FlexScan SX2762W computer display that was 67.4° wide × 41.4° high in visual angle, with a resolution of 2,560 × 1,440 pixels. The display was at a distance of 45 cm from the observer, whose head was held stationary with a head-and-chin rest. The experiment was controlled by an Apple MacBook Air computer.

**Fig. 9:**
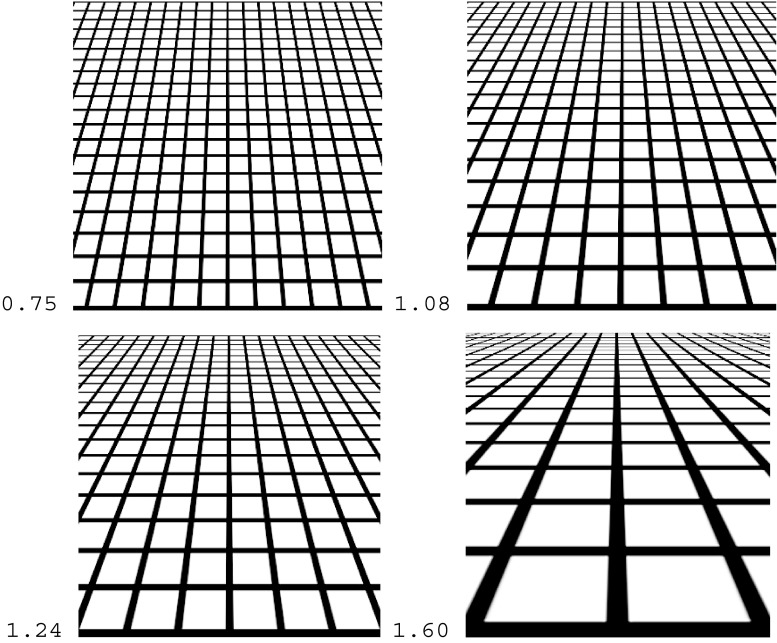
Grid patterns used as the ground pattern. Each pattern was rotated 180° for the ceiling pattern and 90° either clockwise or counterclockwise for the right- and the left-facing-wall patterns, respectively. The number next to each pattern is the steepness of the gradient

The standard stimuli were four grid patterns lacking a difference in lightening (Fig. 9), which were generated by means of Transform in Photoshop. The patterns consisted of black lines of 0.25 cd/m^2^ against a white background of 198.1 cd/m^2^ and were presented against a display of 42.3 cd/m^2^. The patterns were 0.75, 1.08, 1.24, and 1.60 in steepness of gradient, all subtending 32.8° wide × 32.8° high in visual angle. These steepness values approximated those of the flagstone patterns in Experiment 2a. Each pattern was presented in different directions of gradient so as to appear like the ground, ceiling, right-facing wall, and left-facing wall.

To match the apparent slant of each pattern, the G, C, L, and R scales in Fig. 3 were altered so that there was a single movable line in the scale. When the observer scrolled an Apple magnetic mouse forward or backward, the line was changed continuously and simultaneously in orientation and length. When the line was parallel to the frontal plane of the drawn person in each scale (i.e., a slant of 0°), it was shortest in length (6.8° of visual angle), and when the line was maximally slanted 78.0° in depth, it was longest (32.7° of visual angle).

#### Procedure

Each observer sat on a chair with his or her head in a head-and-chin rest. Observation was always binocular with the head upright. The observer first viewed one standard as long as he or she wanted. Meanwhile, the observer was required to register (memorize) the apparent slant of the standard. When the observer was assured of the registration, he or she required the experimenter to present the variable line. After presentation of the variable, the observer adjusted it until it appeared to be the same slant as the standard. The altered G, C, L, and R scales, in each of which the multiple lines were replaced by a variable line, were used for the ground, ceiling, and left- and right-facing wall patterns, respectively. Whenever the observer wanted to see the standard while adjusting the variable, the experimenter switched back to the standard. The observer never saw the standard and the variable simultaneously. The final adjustment of the variable was automatically saved in the computer. For each combination of steepness and direction, two ascending and two descending trials were administrated, so that each observer judged 64 (4 × 4 × 4) combinations, which were randomly presented to each observer.

### Results

**Fig. 10:**
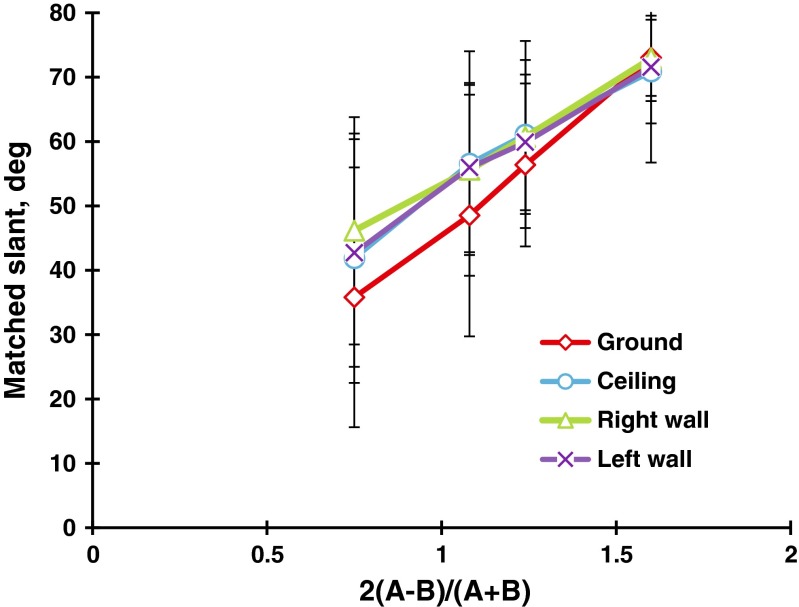
Mean matched slants in degrees for the grid patterns as a function of the steepness of the gradient. The parameter is the direction of the gradient (ground, ceiling, and left- and right-facing walls). The bar attached to each point stands for one standard deviation on either side

For each combination of steepness and direction, four adjustments of the variable were averaged for the statistical scores. Figure 10 shows the mean judged slants taken over 24 observers. A two-way (Steepness × Direction) repeated measures ANOVA revealed that the main effect of steepness was significant, *F*(3, 69) = 57.7, *p* < .001. As is shown in Fig. 10, the mean judged slant increased linearly as the steepness increased. The main effect of direction was significant, *F*(3, 69) = 3.0, *p* < .05, revealing that the mean judged slant of the ground patterns was significantly different from that of the right-facing wall patterns, *t*(69) = 2.8, *p* < .01, with Ryan’s test.

The Steepness × Direction interaction was significant, *F*(9, 207) = 2.8, *p* < .01. The simple main effects of direction were significant for the steepnesses of 0.75, *F*(3, 276) = 5.7, *p* < .001, and 1.08, *F*(3, 276) = 4.4, *p* < .01. Ryan’s tests indicated that for the steepness of 0.75, the mean judged slant of the ground pattern (35.8°) was significantly different from those of the ceiling pattern (41.8°), *t*(276) = 2.4, *p* < .05; the right-facing wall pattern (46.1°), *t*(276) = 4.0, *p* < .001; and the left-facing wall pattern (42.7°), *t*(276) = 2.7, *p* < .01. For the steepness of 1.08, the mean judged slant of the ground pattern (48.5°) was again significantly different from those of the ceiling pattern (56.6°), *t*(276) = 3.1, *p* < .01; the right-facing wall pattern (55.6°), *t*(276) = 2.8, *p* < .01; and the left-facing wall pattern (55.9°), *t*(276) = 2.9, *p* < .01. *MSE* = 78.8 for all subordinate tests.

### Discussion

**Fig. 11:**
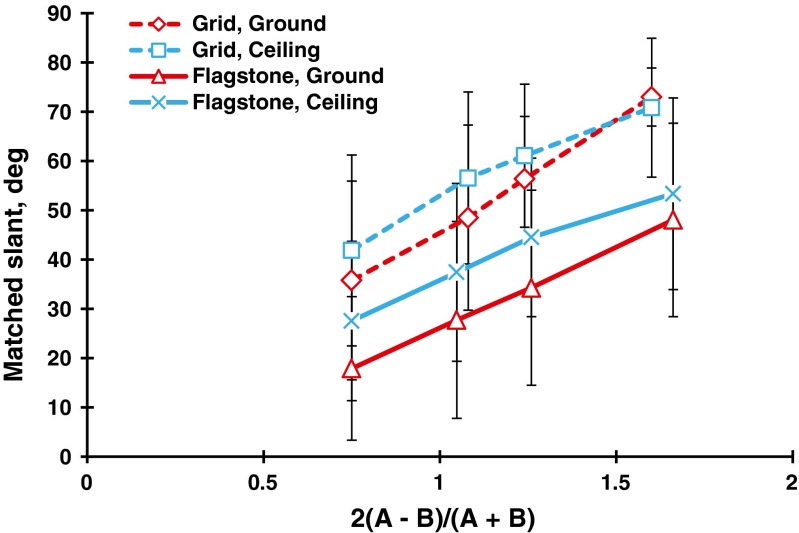
Comparison between the ground and ceiling patterns for the grid and flagstone patterns in Experiments 2a and 2b. The bar attached to each point stands for one standard deviation on either side

To compare the mean judged slants of the flagstone textures in Experiments 2a to those of the grid textures in Experiment 2b, we have superimposed the results of the ground and ceiling patterns in Fig. 11. Clearly, there were anisotropic slant effects for both the grid and flagstone patterns. Specifically, for the grid textures, the differences in mean judged slant between the ground patterns and the other patterns were about −1° to 8°, which were almost the same as those of the polka-dot patterns in Experiment 1 (i.e., about 0° to 6°). For the flagstone textures, these differences were about 10° to 14°, which correspond to about 5° to 13° for the flagstone textures in Experiment 1. Thus, the anisotropic slant effects may depend on the texture element but not on the presence/absence of the HMD or the method of measurement. Incidentally, the mean judged slants of the grid patterns were larger than those of the flagstone patterns. We will later consider the differences among the polka-dot, grid, and flagstone patterns.

## Experiment 3

**Fig. 12:**
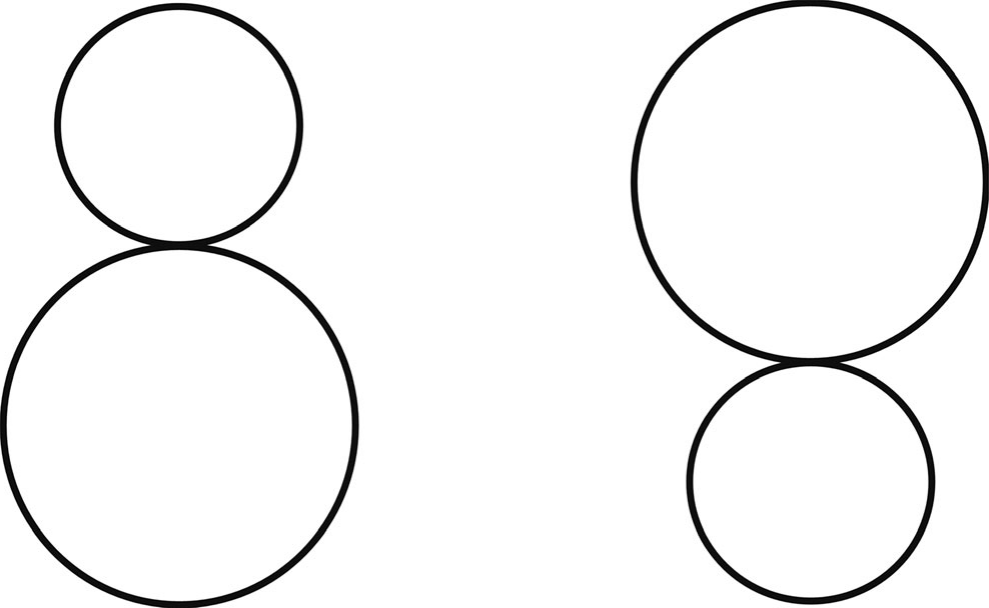
Size contrast illusion depending on the relative locations of circles

The ground patterns appeared closer to the frontal plane than the ceiling and sidewall patterns. How could this type of anisotropy be explained? The subsequent experiments were concerned with this problem. One possible explanation was based on the tendency to perceive the size of the upper part of visual space to be larger than the size of the lower part (Luckiesh, 1922, p. 44; Robinson, 1972, p. 104). Figure 12 illustrates this tendency, in which the difference in size between the circles in the left half is perceived to be smaller than that in the right half. This optical illusion should be called the *positional effect of size contrast*. We assumed that the anisotropic slant effects found in Experiments 1 and 2 are the accumulated positional effects of size contrast. That is, the ceiling patterns would appear steeper in the gradient of their element sizes and more slanted than the ground patterns. To examine this prediction, in Experiment 3, the same-sized dots were distributed regularly in a display with different gradients of dot density. In other words, the gradient of dot size was eliminated, but the gradients of interdot interval and dot density and the linear perspective made by dotted lines were retained. We assumed that these renewed patterns would be weaker in positional effects of size contrast than the polka-dot, grid, and flagstone patterns of Experiments 1 and 2.

### Method

#### Observers

The observers were 23 university students (six males and 17 females) with a mean age of 20.1 years.

#### Apparatus and visual patterns

**Fig. 13:**
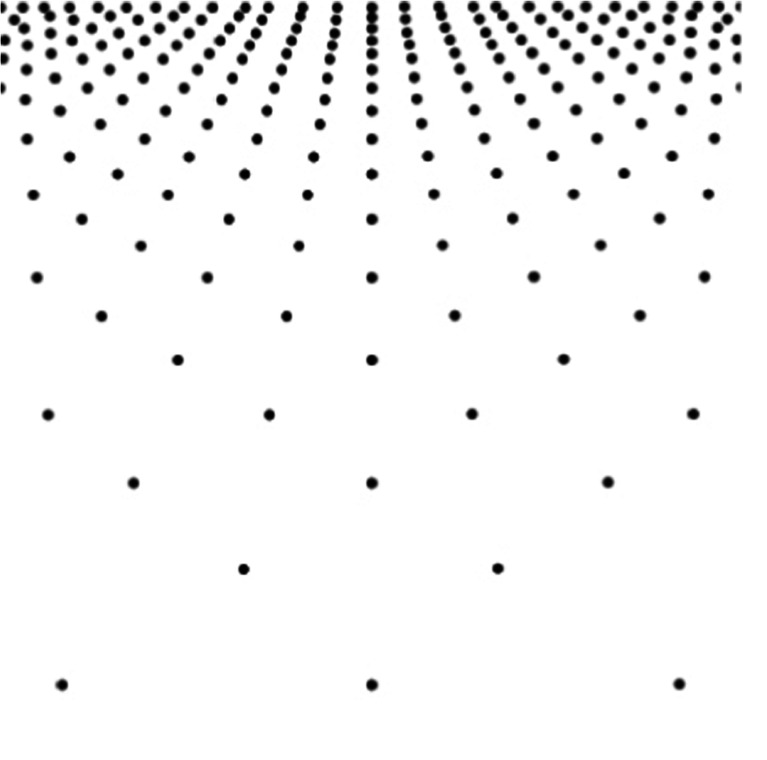
Regularly arranged same-sized-dot pattern used as the ground plane. This pattern was rotated 180° for the ceiling pattern, and 90° either clockwise or counterclockwise for the right- or left-facing wall pattern, respectively. The steepness of the gradient is 1.44

The apparatus was the same HMD used in Experiment 1. The five patterns consisted of numerous dots of 0.5° diameter each (Fig. 13) that were substituted for the polka dots in Experiment 1. Each pattern was 32.8° wide × 32.8° high in visual angle, and the steepness values were 0.0, 0.49, 0.86, 1.24, and 1.44. These patterns were presented in different directions of gradient, so that the dot density increased from bottom to top (ground), from top to bottom (ceiling), from right to left (left-facing wall), and from left to right (right-facing wall). To match the apparent slant of each pattern, the G, C, L, and R scales shown in Fig. 3 were presented 1.1° to the right of the pattern within the HMD.

#### Procedure

After having put on the HMD, each observer held his or her head forward and viewed one pattern at a time binocularly. The observer was asked which of the higher or lower density of each pattern was farther away from the observer and was then required to choose one line or two neighboring lines that were most suitably matched to the slant of the textured plane. The G, C, L, and R scales were used for the ground, ceiling, and left- and right-facing wall patterns, respectively. Each observer judged 40 (5 × 4 × 2) combinations of steepness, direction, and replication, which were randomly presented for each observer.

### Results

**Fig. 14:**
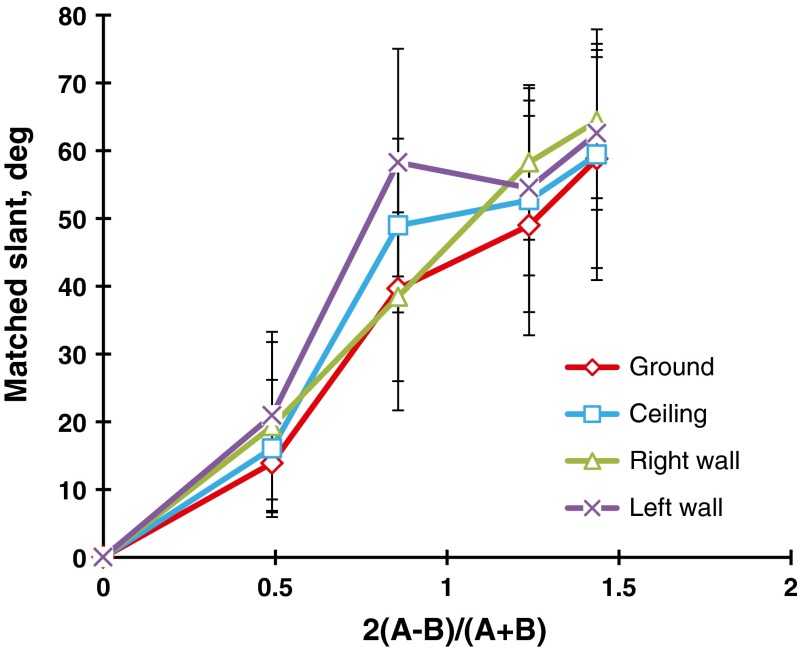
Mean matched slants in degrees for the regularly arranged same-sized-dot patterns. The parameter is the direction of the gradient: ground, ceiling, and sidewalls. The bar attached to each point stands for one standard deviation on either side

The observers’ judgments were converted into slants in degrees as before, and the two replications were averaged for the statistical scores. Figure 14 shows the mean judged slants taken over 23 observers. A two-way (Steepness × Direction) repeated measures ANOVA revealed a main effect of steepness, *F*(4, 60) = 398.1, *p* < .001: The mean judged slant increased in a zigzag way as the steepness of the gradient increased.

The main effect of direction was significant, *F*(3, 60) = 11.5, *p* < .001, and the Steepness × Direction interaction was also significant, *F*(12, 264) = 8.7, *p* < .001. For the steepness values of 0.49, 0.86, and 1.24, the simple main effects of direction were significant, *F*(3, 330) = 4.1, 34.6, and 6.1, respectively, *p* < .01 for all tests. Ryan’s tests were performed for each of the significant steepness levels. For the steepness of 0.49, we found a significant difference in mean judged slants between the ground (13.9°) and left-facing wall (20.9°) patterns, *t*(330) = 3.2, *p* < .05. For the steepness of 0.86, significant differences emerged between the ground (39.6°) and ceiling (49.0°) patterns, *t*(330) = 4.2, *p* < .05; between the ground and left-facing wall (58.3°) patterns, *t*(330) = 8.4, *p* < .05; between the right-facing wall (38.4°) and ceiling patterns, *t*(330) = 4.7, *p* < .05; between the ceiling and left-facing wall patterns, *t*(330) = 4.2, *p* < .05; and between the sidewall patterns, *t*(330) = 8.9, *p* < .05. For the steepness of 1.24, we observed a significant difference between the ground (49.0°) and right-facing wall (58.3°) patterns, *t*(330) = 4.2, *p* < .05. *MSE* = 56.7 for all subordinate tests.

Thus, for the regular same-sized-dot patterns with a moderate steepness of gradient, the mean judged slants of the ground patterns were the same as or less than those of the ceiling or sidewall patterns, but the tendency to judge the ground patterns to be less slanted was not as definite as in the results of Experiments 1 and 2.

## Experiment 4

One may ask why the anisotropic slant effects were not largely reduced in Experiment 3. An interpretation would be that even when the gradient of dot size was eliminated, the gradients of interdot interval and dot density and the linear perspective of dotted lines remained. In Experiment 4, we attempted to remove these residual sources of information that might potentially be available to generate size contrast. For this purpose, we used dynamic random-dot patterns with different gradients of dot velocity. In each pattern, all of the dots moved in the same direction to form a gradient of dot velocity. The time that each dot was lit on was random but the duration until it was lit off was constant, so the mean interdot interval and the mean dot density were the same over the display, and there was no linear perspective of dotted lines.

It is clear that the gradient of velocity is effective for producing slant perception (Braunstein, 1976; Gibson, 1950b), but the relative contribution of dynamic versus static textures is not very clear. Braunstein (1976) showed that when velocity information is combined with static texture information in judging slant, the weight given to velocity information was more than twice that given to the static texture information. Cornilleau-Pérès et al. (2002) showed that when motion parallax as a depth cue was in conflict with a static grid, the slant indication coming from the grid dominated the slant indication from motion parallax. In either case, it was expected that the anisotropic slant effects would be more weakened or would be eliminated in this experiment.

### Method

#### Observers

The observers were 22 university students (eight males and 14 females) with a mean age of 20.2 years.

#### Apparatus and visual patterns

The apparatus was the same HMD used in Experiment 1. The dynamic patterns were composed of 200 white random dots with a diameter of 0.875° of visual angle each and with a luminance of 67.45 cd/m^2^ against a black background of 1.45 cd/m^2^. The patterns were 30.6° wide × 30.6° high in visual angle and were presented straight ahead of the observer. All of the dots in each pattern moved in the same direction with a gradient of velocity, and each dot was lit on at a time and place determined randomly and then moved for 167 ms until it was lit off. Seven gradients of dot velocity were created, in which the differences between the fastest and slowest dot velocities were 0°/s (i.e., 52.5 – 52.5), 17.5°/s (61.3 – 43.8), 35.0°/s (70 – 35), 52.5°/s (78.8 – 26.3), 70.0°/s (87.5 – 17.5), 87.5°/s (96.3 – 8.8), and 105°/s (105 – 0). The mean velocity for all patterns was 52.5°/s. The steepness of gradient for the dynamic patterns was defined as 2(*A* – *B*)/(*A* + *B*), where *A* is the fastest velocity of the dots and *B* is the slowest one. This definition corresponds to the definition for the gradients of static elements. The steepness values used here were 0.00, 0.33, 0.67, 1.00, 1.33, 1.67, and 2.00. Each pattern was then presented so as to appear as the ground (i.e., fastest velocity at the bottom and slowest velocity at the top), the ceiling (fastest velocity at the top and slowest velocity at the bottom), or a sidewall (fastest velocity at the left and slowest velocity at the right, or vice versa). For the ground and ceiling patterns, the dots were moved from left to right or right to left, whereas for the sidewall patterns, the dots were moved from top to bottom or bottom to top. To match the apparent slants of each pattern, the G, C, L, or R scale was presented 1.1° to the right of each pattern within the HMD.

#### Procedure

After having put on the HMD, each observer held his or her head forward and viewed one pattern at a time binocularly. The observer was asked which of the faster or the slower velocities in each pattern were farther away, and the observer was then required to choose either one line or two neighboring lines that were most suitably matched to the slant of the dynamic surface. The G, C, L, and R scales were used to match the apparent slants of the ground, ceiling, and left- and right-facing wall patterns, respectively. Each observer judged 56 (7 × 4 × 2) combinations of gradient steepness, gradient direction, and motion direction, which were randomly presented for each observer.

### Results

**Fig. 15:**
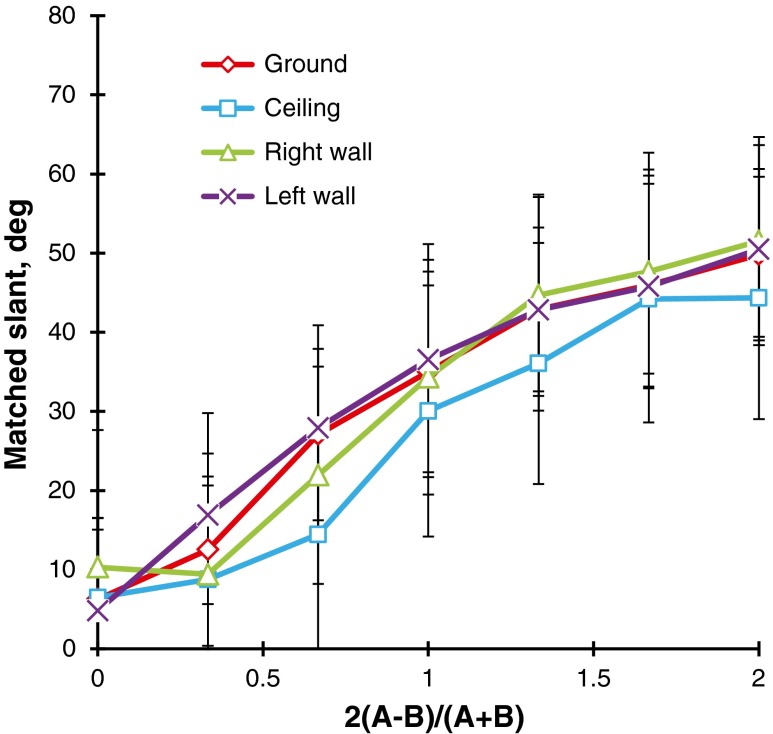
Mean matched slants in degrees for the dynamic random-dot patterns with gradients of velocity. The parameter is the direction of the gradient (ground, ceiling, and sidewalls). The bar attached to each point stands for one standard deviation on either side

The observers’ judgments were converted into slants in degrees as before. The slants of the two motion directions were averaged for the statistical scores. Figure 15 shows the mean judged slants taken over 22 observers. A two-way (Steepness × Gradient Direction) repeated measures ANOVA was performed. The main effect of steepness was significant, *F*(6, 126) = 129.7, *p* < .001, indicating that the mean judged slants increased curvilinearly as the steepness of the gradient increased. The main effect of direction was significant, *F*(3, 63) = 3.8, *p* < .05. Ryan’s tests indicated that the ceiling pattern was judged to be less slanted than the ground, *F*(66) = 2.5, *p* < .05; left-facing, *F*(66) = 3.0, *p* < .05; and right-facing, *F*(66) = 2.6, *p* < .05, wall patterns. *MSE* = 293.4 for all subordinate tests.

The Steepness × Direction interaction was significant, *F*(18, 378) = 1.9, *p* < .05. For steepness values of 0.33, 0.67, and 1.33, the simple main effects of direction were significant, *F*(3, 441) = 2.7, 7.6, and 2.8, respectively, *p* < .05 for all tests. Ryan’s tests were performed for each of the significant steepness levels. For the steepness of 0.33, no pattern was significantly different in mean judged slant from the other patterns. For the steepness of 0.67, the mean slant of the ceiling pattern (14.5°) was significantly different from those of the ground pattern (27.1°), *t*(441) = 4.0, *p* < .05; the right-facing wall pattern (21.9°), *t*(441) = 2.4, *p* < .05; and the left-facing wall pattern (27.9°), *t*(441) = 4.3, *p* < .05. For the steepness of 1.33, the mean judged slant of the ceiling pattern (36.1) was significantly different from that of the right-facing wall pattern (44.7°), *t*(441) = 2.7, *p* < .05. *MSE* = 110.1 for all subordinate tests.

Thus, in four cases, the *ceiling* patterns were here judged to be less slanted than the other patterns, and in the other cases, no significant difference was apparent among the directions of gradient. In no case were the ground patterns judged to be less slanted than the other patterns.

## General discussion

In Experiments 1 and 2, slanted surfaces were produced by regular patterns of polka dots, grid lines, or flagstones. These elements created gradients of element size, interelement interval, and element density, as well as the linear perspective made of the connected elements. When these patterns were observed with different directions of gradient, the ground patterns were judged to be less slanted from the frontal plane than were the ceiling and sidewall patterns. This tendency was independent of head position. To be exact, the ground patterns had a strong tendency for the surface to appear to be more frontal to the Frankfort line than it was. This head-centric slant effect should not be confused with the optical slant effects that have been referred to by Gibson (1950a) and Durgin, Li, and Hajnal (2010).

Several previous studies had failed to find anisotropic slant effects. Gibson (1950a) noted that the ground pattern was judged to be less slanted than the ceiling pattern, but he attributed this to a constant error that intruded into the matching procedures. We believe, however, that anisotropic slant effects are perceptual, because the same effects were obtained by means of different comparison modes and response modes (Exp. 2). Rosas et al. (2004, their Exp. 2) generated their ground and sidewall surfaces by using circle or Perlin-noise texture gradients and determined a difference threshold for each surface, but they did not find a clear difference in thresholds between the directions of gradient. However, their number of observers (*N* = 2) was too small to achieve a less biased outcome.

We assumed that anisotropic slant perception may be the result of accumulated positional effects of size contrast, and also assumed that if the size contrast were reduced, the anisotropy might be reduced. To reduce size contrast, in Experiment 3 the gradient of dot size was eliminated by holding dot size constant, but the anisotropy was still detected in the judged slants. To furthermore reduce size contrast, in Experiment 4 same-sized dots were moved in the same direction with gradients of velocity. This meant that the gradients of dot size, interdot interval, and dot density and the implicit linear perspective were replaced by a gradient of velocity. In this experiment, the anisotropy mentioned above was not detected, and the ceiling patterns were sometimes judged to be less slanted than the others. These results may partially support the size contrast hypothesis.

Li and Durgin (2013) failed to find directional effects of the texture gradient of a surface on apparent relative extent. The observers viewed a grassy texture under an immersive virtual environment and judged the ratios between the frontal and in-depth extents. When the same textured surface was slanted in uphill, downhill, leftward-slanted, and rightward-slanted directions, the relative judgments did not differ among the texture directions. This result seems to have mainly been due to the very fine grassy elements that constituted the surface, which would not induce a sufficient size contrast among the elements.

When texture elements varied regularly in size, as in Experiments 1 and 2, the judged slant of the textured surface increased linearly as the steepness of the gradient increased, but when the texture elements were held constant in size, the relation of judged slant to the steepness seemed to deviate from linearity, as can be seen in Figs. 14 and 15. This suggests that the gradient of element size is an important source of information for judging the slants of textured surfaces. Cutting and Millard (1984) indeed showed that both perspective and the compression of texture elements are more effective when judging the slant of flat surfaces than the gradient of density. Similar arguments were made by Braunstein (1976) and Durgin (1995, his Fig. 11).

Other explanations of the anisotropic slant effects are possible. One explanation is based on the relation of surfaces to the horizontal line. One of the surfaces that we most frequently encounter is the ground, which has the largest surface in the visual world and intersects with the horizontal line at eye level. So, we are apt to perceive the ground surface as rising up at the far distance. On the other hand, the ceiling surface is usually parallel to the ground but is so short that we are not aware that it would appear to hang down to the horizon: We are apt to see the ceiling surface as being flat and parallel to the ground. The sidewall surfaces are perceived to converge at the far distance, but they do not have a line like the horizontal line to which the ground and ceiling surfaces converge. From these observations, it may be assumed that the surface that closely pertains to the horizontal horizon line would appear much closer to the frontal plane. This hypothesis predicts that the ground pattern should appear to rise up steeply at the farther distance, the ceiling pattern should appear to hang down moderately, and the sidewall patterns should appear to extend almost straight. The present results may support this prediction as a whole.

Several aspects of our results, however, may contradict the horizontal-line hypothesis. First, as was indicated by the results of Experiment 1, the mean judged slants did not change with head position. If the horizontal-line explanation were correct, the textured surfaces that were observed with the head forward would be perceived to be less slanted than those that were observed with the head upward or downward. Second, if this explanation were correct, the judged slants of the ground pattern would be the same, regardless of whether the texture elements were static or dynamic.

Recently, Bian and colleagues (Bian & Andersen, 2008; Bian et al., 2005, 2006) have found that to organize 3-D scenes, we make use of optical contact information provided by the ground, not by the ceiling or sidewalls. Their finding seems to support Gibson’s (1950b) ground theory, which emphasizes the importance of the ground surface in space perception. However, we do not know how the anisotropic slant effects are related to the organization of objects on the ground.

The anisotropic slant effects may contradict the predictions from many empirical theories of distance perception (see Ross & Plug, 2002, chap. 9, for a review). In these theories, it is generally assumed that we are very familiar with the ground through visual and haptic experiences, so that horizontal distance is well discriminated and is perceived to be longer than the distances in other directions. This explanation may be applied to comparison of the ground and ceiling surfaces: In everyday life, there are a large number of objects on the ground that we can see and touch, whereas on the ceiling are a small number of objects that we can see but cannot touch. Because of this difference, the ground surface may appear greater in depth than the ceiling surface. On the basis of a simple geometric principle, this implies that if the ground and ceiling surfaces subtended the same visual angle at the same viewing distance, the ground surface would appear more slanted from the frontal plane than the ceiling surface.

The empirical distance theories may come back into existence by assuming that perceptual and judgmental levels are situated in the visual system. That is, it is assumed that (1) objects on the ground are perceived to be farther away and larger through the size–distance invariance than are those on the ceiling, but (2) larger perceived objects are judged to be closer according to the size–distance paradox, and (3) the ground surface on which the objects are placed is judged to be less slanted. This explanation does not seem persuasive, because the size–distance paradox has been observed for very far objects, such as the sun and the moon (Hershenson, 1989; Ross & Plug, 2002), or for monocularly viewed close objects for which only accommodative vergence is available as a distance cue (Heinemann, Tulving, & Nachmias, 1959; Higashiyama, 1979; Ono, Muter, & Mitson, 1974). It should be recalled that in this study, accommodation, binocular convergence, and binocular disparity were in conflict with various types of texture gradients. These viewing conditions may differ from situations that induce the size–distance paradox. In addition, it may be difficult to test the multiple stages of processing that are assumed by the empirical distance theories. Under the current status of the data collected, the size contrast hypothesis is much simpler than the empirical distance theories.

**Fig. 16:**
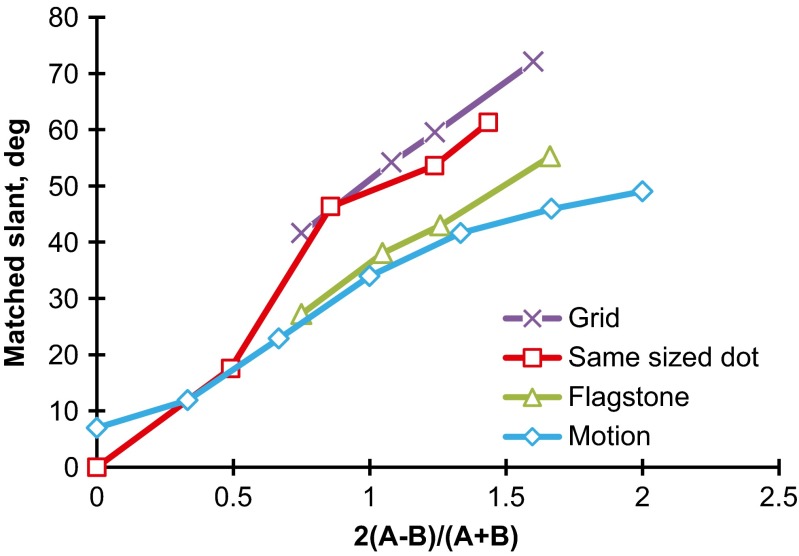
Comparison of the mean matched slants in degrees as a function of the steepness of the gradient among the grid (Exp. 2b, *N* = 24), same-sized-dot (Exp. 3, *N* = 23), flagstone (Exp. 2a, *N* = 21), and motion (Exp. 4, *N* = 22) patterns. Each data point is the mean of the four directions of gradient

It may be useful to note the differences in judged slant among the grid, same-sized-dot, flagstone, and motion patterns. Figure 16 shows the mean judged slants for the four patterns taken over the four directions of gradient. For a steepness of 0.5 or less, the mean judged slants were almost the same for the four patterns, but for a steepness of 1.0 or more, the mean judged slants for the static patterns were larger than those for the motion patterns. This result may agree with the Cornilleau-Pérès et al. (2002) study: Static information is more useful to generate apparent slant than is velocity information.

Figure 16 also shows that the mean judged slants of the grid and same-sized-dot patterns were larger than those of the flagstone patterns. This confirms the results of Experiment 1, in which the polka-dot patterns were more slanted than the flagstone patterns. The difference between the geometric and natural textured surfaces is the lightness contrast between the texture elements and the background, which may affect discrimination of the texture. Compare the ground patterns in Figs. 2 and 9 that have almost the same steepness of gradients. Clearly, the grid patterns are higher in lightness contrast than are the flagstone patterns. The grid lines can be perceived separately even when the texture density is high, whereas the flagstones are fused at the same place. The fused elements may weaken the effects of texture gradient on apparent slant (Gruber & Clark, 1956).

Finally, the question still remains of why the ceiling patterns were sometimes judged to be less slanted than the other patterns in Experiment 4, in which all possible sources of size contrast were eliminated. It is difficult to interpret this finding, but it has been found that when irregular arrays of many small and simple geometric elements were slanted by shear disparity, “the ground surface appeared inclined from the vertical between 12° and 15° more than the ceiling surface” (Pierce, Howard, & Feresin, 1998, p. 98). However, we do not know how the present result is related to the anisotropy of stereoscopic slant.

To sum up, we have demonstrated that there is visual anisotropy in slant perception, in that the slant of static textured planes depends on the direction of the gradient. Specifically, ground patterns are judged to be less slanted than ceiling and sidewall patterns. This anisotropy occurred for size gradients of static elements but not for velocity gradients of dynamic elements. These effects were interpreted to be the accumulated positional effects of size contrast among the texture elements.
